# Arbor: Comparative Analysis Workflows for the Tree of Life

**DOI:** 10.1371/currents.tol.099161de5eabdee073fd3d21a44518dc

**Published:** 2013-06-21

**Authors:** Luke J. Harmon, Jeffrey Baumes, Charles Hughes, Jorge Soberon, Chelsea D Specht, Wesley Turner, Curtis Lisle, Robert W. Thacker

**Affiliations:** Synthetic Reality LaboratoryUniversity of Central Florida; University of Kansas; University of California, Berkeley; Medical VisualizationKitware, Inc.; KnowledgeVis, LLC; University of Alabama at Birmingham

## Abstract

We describe our efforts to develop a software package, Arbor, that will enable scientific research in all aspects of comparative biology. This software will enable developmental biologists, geneticists, ecologists, geographers, paleobiologists, educators, and students to analyze diverse types of comparative data at multiple phylogenetic and spatiotemporal scales using an intuitive visual interface. Arbor’s user-defined workflows will be exported and shared so that entire analyses can be quickly replicated with new or updated data. Arbor will also be designed to easily and seamlessly expand to include novel analytical tools as they are developed. Here we describe the core components of Arbor, as well as provide details of one proposed test case to illustrate the software’s key functionality.

## Introduction

Scientists are making rapid progress building the Tree of Life, which promises to provide new insights into evolutionary relationships across space and time. We now have available large-scale phylogenies (e.g. 1 2) and are accumulating massive amounts of character and distributional data (e.g. plant traits 3; mammalian traits 4; species occurrence data 5; gene interactions 6; microbiomes 7). Together, these resources provide researchers with unique opportunities to investigate processes that drive biological diversification at broad and deep phylogenetic scales (e.g. 8 9 10). Developing tools that enable comparative analysis of various types of character data across large-scale phylogenies is essential to further our understanding of fundamental evolutionary processes.

As a “first draft” of the Tree of Life emerges, it becomes increasingly clear that we lack the resources necessary to interpret the evolution of character data across phylogenies at large scales. This is largely due to a lack of tools for the integration and visualization of massive amounts of spatial, temporal, and character data and for placing this information into the comparative framework provided by phylogenetic trees 11. Comparative analyses are hindered by the lack of scalable programs for studying trait evolution, the heterogeneity of data formats, and the inability to integrate multiple analyses to enable novel discoveries 12. Tools that successfully integrate very large phylogenetic trees with vast amounts of data associated with branch tips and nodes will enable evolutionary biologists to visualize and investigate the processes that drive the evolution of organismal diversity.

Here we present an initial description of a new open-source software package, Arbor, which is currently in development. Funded by the NSF AVAToL program, Arbor will allow us to harvest massive amounts of information from phylogenetic trees to facilitate new discoveries about the evolution of life on Earth.

## Arbor: a new vision for comparative analyses

To bring comparative biology up to speed with the emerging Tree of Life, we are developing Arbor**, **an extendable platform for analyzing and visualizing evolutionary processes in a phylogenetic framework. Using Arbor workflows, researchers will be able to investigate evolutionary questions across very large phylogenetic trees containing very large numbers of terminal units (e.g. species, populations, individuals) using diverse types of character data (e.g. traits, biogeography, ecological associations, species interactions). The goal of Arbor is to provide biologists with an immediate and intuitive interface for testing comparative evolutionary questions across the Tree of Life and to enable them to quickly and efficiently communicate their findings to scientific and general audiences.

We are currently developing and implementing flexible and extendable tools that interact through an intuitive visual workspace in Arbor. As designed, Arbor workflows have three main benefits. First, Arbor will be flexible: the workspace allows end users to link together custom-designed sets of statistical analyses geared toward the types of questions inherent to their data. Second, Arbor will be extendable: software developers are able to quickly and easily add functionality without altering the details of Arbor’s core implementation. Third, Arbor will be sharable: users will be able to save their Arbor workflows and share them with others. As such, Arbor will be “next generation” software that enables tree-thinking and novel analyses at broad spatial and temporal scales.

The Arbor development team (see below) brings together a variety of expertise in both biology and computer science. The ongoing development of Arbor focuses on four components. First, we are developing the “core” of Arbor. This core will be a phylogenetic comparative analysis platform with a flexible user interface that will leverage existing software, algorithms and web services to allow scientists to address comparative evolutionary hypotheses at every scale across the Tree of Life. Second, we are implementing shared data integration and basic analysis tools for Arbor. These modular tools will provide basic visualization and fast, scalable algorithms for comparative analyses across a given phylogeny (i.e. lineage-specific trait diversification) or between two topologies (i.e. co-diversification, species interactions). Third, we are using a set of three test cases to develop Arbor into software that can solve fundamental problems in evolutionary biology. These test cases will stimulate rapid development of numerous Arbor modules that are directly inspired by biological applications across the Tree of Life. Fourth, we are developing four outreach initiatives to ensure that Arbor has a broader impact in advancing science, education and public education in comparative evolutionary biology.

## The Arbor core: workflows and web services promote flexible and dynamic analyses

The Arbor interface will be a radical departure from currently existing software for comparative analyses. Users will interact with Arbor in an intuitive, visual way; building an Arbor workflow will have more in common with building a structure using Legos than with programming a computer. By using a modular open-source interface and web services, we will encourage the development of new tools and simultaneously enhance interoperability among existing tools.

Arbor takes inspiration from existing workflow software, especially ParaView 13 (also see Galaxy 14 and the iPlant Discovery Environment^33^). Users of workflow software create problem-specific workflows visually by connecting a series of data manipulations and analyses in a series of steps. The underlying software system is able to automatically coordinate the invocation of all of these steps during workflow execution (see the test case described below for an example workflow design).

We are designing Arbor as scalable software, with tree-of-life-scale analyses in mind from the beginning. Arbor will be able to efficiently process both small and large datasets, ultimately extending to analyses across the entire Tree of Life. This scalable performance will come from the use of improved algorithms (e.g. 15) along with both fine-grained parallelism (using multithreaded execution of individual steps when possible in a workflow) and coarse-grained parallelism (executing multiple steps of a workflow simultaneously on separate cores). Arbor will run efficiently to develop and test workflow designs using small and medium-size datasets on laptop and personal computers. Then, after a workflow idea has been expressed and validated, the workflow can be run against tree-of-life scale data using a scalable computing resource. To do this, the user will transfer the workflow from the local computer to an Arbor instance running on a large, multiprocessing computer, a computation cluster (e.g., XSEDE; www.xsede.org), or a similar cloud computational service to run a full-scale analysis.

Arbor will be modular software, extendable through a plug-in architecture that allows new analysis modules to be added to the program (similar to the modular design of Mesquite 16). Arbor modules will be able to include algorithms written in a number of languages (e.g. R, Python, Perl, C, or C++). Researchers who develop software for comparative analyses will have an immediate user base if they integrate their program as an Arbor module, promoting and encouraging active use of Arbor by the programming community. Arbor’s modular construction will allow new algorithms to become quickly available and usable with data; scientists will be able to carry out comparative analyses that have not yet been imagined.

Instead of spending effort redeveloping yet another implementation of known algorithms, the Arbor team will focus initially on integrating with existing external applications. Arbor will be able to work alongside proven systems like Mesquite, MrBayes, BEAST, PHYLIP, ape, and others. At any point along the way, Arbor’s visualization tools can be used to examine intermediate steps along an analysis workflow. All of the analyses in a workflow will be coordinated through the Arbor interface. This integration represents an entirely new way to build novel comparative analyses.

Arbor will include a web services infrastructure, providing the ability to integrate with existing web-hosted services that offer trees and data for analysis or perform algorithms useful for Arbor workflows (e.g. Lifemapper, CIPRES, MAFFT, RAXML, UniFrac, TreeBase, PorToL, OneZoom, and iPLANT). We will also make specific connections to the other AVAToL projects (Open Tree of Life and Next-Generation Phenomics). These connections will be facilitated by Arbor’s *dataIntegrator* module, which will allow Arbor to access, integrate, and analyze phylogenetic, geospatial, ontogenetic, and morphological data together in a single workflow.

Users will be able to export and share their workflows so that entire analyses can be quickly replicated with new or updated data. Shared workflows will be stored at the DRYAD digital repository (datadryad.org) or a similar archive, so that users who generate practical or compelling workflows receive direct credit via citations in scientific publications. We will encourage buy-in from user communities by providing an interactive and intuitive visual exploratory workspace with a user-driven toolkit. This toolkit can be expanded to include novel analytical tools as later modules are developed to serve the scientific community. Furthermore, we plan to quantify the impact of Arbor in real time. Tool use could be tracked to demonstrate user action and efficiency, providing feedback on which programs are being used and combined. Workflows will capture the provenance of ideas by automatically generating a list of references to be cited by end-users.

## What will I be able to do with Arbor?

Arbor will begin by implementing the most commonly used existing comparative algorithms. We have started from the most flexible current platform for comparative methods, the statistical computer programming language R, home to several widely used phylogenetic analyses packages including ape 17, GEIGER 18, picante 19, and diversitree 20. The comparative algorithms from these packages – especially ape – will form the initial basis for Arbor’s core analysis infrastructure. We will also focus on developing and implementing new algorithms that can be applied efficiently to very large datasets (e.g. 15).

Arbor will allow users to link analyses as a series of steps in a workflow. To facilitate this process, we will spend effort to extend the flexible and scalable data structures already implemented in ParaView and/or VTK (the Visualization Toolkit 22) to be useful for a wide array of evolutionary analyses. Phylogenetic tree data structures are initially based on designs implemented in the ape and phylobase R packages. Our project will study and refine the design of tree and data structures for scalability and efficiency.

We will include powerful tree-based selection and query operations in Arbor’s analytical capabilities through the *treeManipulator* module. For example, Arbor will be able to locate sets of species that co-occur at a particular place and time, and to plot those species on a phylogenetic tree. This type of analysis, combining phylogenetic, geospatial, temporal, and other evolutionary traits, will take advantage of the rich filtering and selection operations implemented in Arbor’s analysis framework. These steps can then be chained together to form problem-specific workflows.

A strength of Arbor will be the *phyloVis* module, which will use a mature set of interactive graphical tools already extensively tested by the scientific visualization and data-mining communities. VTK-based visualizations have previously been implemented in interactive user interfaces running on all major computing platforms (Windows, Mac, Linux, and various custom supercomputer architectures). The VTK library itself is open-source and has been steadily evolving and expanding since its initial releases. In the past few years, the Visualization Toolkit has been extended to include informatics and information visualization tools to VTK, making VTK a premier information visualization platform that includes pre-built interactive visualization views 23. The *phyloVis* module will take advantage of these views to include visualizations specific to comparative analyses. We will also allow users to interface with OneZoom, a new tool for visualizing very large phylogenetic trees 11.

We will employ a set of three test cases to develop Arbor into software that can address fundamental questions in evolutionary biology. Test cases have been selected for their overarching biological relevance, but also to deliberately challenge and drive software development. These test cases include: (I) *The Evolutionary Process of Spatial Diversification*, using species distribution data, phylogenetic relationships, and temporal data to understand processes underlying biogeographic patterns and ecological niche differentiation; (II) *The Evolution of Symbiotic Communities*, using natural evolutionary replicates to understand the tempo and mode of evolution in species interactions and the evolution of phylogenetic community structure; (III) *The Evolution of Complex Interactions*, using novel evolutionary models and analytical algorithms to understand functional diversification during macroevolution and the evolution of interaction networks. Below we describe one test case in more detail to give an idea of the potential of the Arbor framework for enabling novel analyses.

## Arbor Test Case: The Evolutionary Process of Spatial Diversification

The geographic range of a species is a crucial feature of its evolution. Species’ ranges are determined by both contemporary and historical factors 24. Currently, species distribution models (SDMs) focus on the impact of contemporary abiotic factors in structuring species’ ranges 25. By contrast, biogeographic models (such as Lagrange 26) consider how species’ ranges change through time due to dispersal, speciation, and extinction 27 28. Arbor will allow users to integrate both approaches to allow comprehensive analyses of the evolution of species’ ranges over time.

For this test case, we will combine locality data with dated phylogenetic trees and paleoclimate data to model species distributions over time. Our test case analyses will focus on two main goals: (I) including historical data in SDMs; and (II) including information about species’ niches in biogeographic models.

To include historical data in Species Distribution Models (SDMs), the Arbor data integration module (*dataIntegrator*) will query occurrence data (e.g, VertNet [vertnet.org], GBIF [www.gbif.org], iDigBio [www.idigbio.org], or others) to obtain locality information for individuals from many species within a clade, and climate data servers to obtain climate data for each locality. An intuitive visual output will enable the user to effectively curate occurrence data, selecting data points that are relevant for the analysis. A distribution model for each of these species using the curated occurrence data will be constructed using an *openModeller* module (based on openmodeller.sourceforge.net) and LifeMapper (lifemapper.org), an existing set of web services and tools. *openModeller* will use the integration capabilities of Arbor to incorporate both current distribution data and the phylogenetic relationships of species into SDMs. Using these data, the *openModeller* module will be able to project species ranges onto past climatic layers using species’ distribution models combined with paleoclimatic data, given the phylogenetic relationships of the species and the temporal scale of their ancestral distributions. The results can then be visualized using the *phyloVis* module.

**Proposed phyloFlow workflow for the test case developed in the text. Information processing flows from remote sources to five phyloFlow modules.**
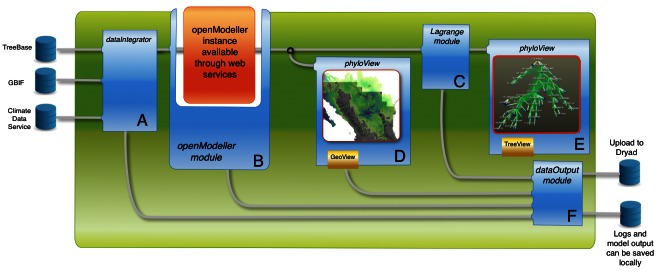
First, the dataIntegrator (A) combines phylogenetic, distributional, and climactic data sources. openModeller (B) then creates species distribution models, and Lagrange (C) tests historical biogeographic hypotheses incorporating those models. Next, phyloView allows geographic (D) and phylogenetic (E) visualization of results. Outputs are directed to the dataOutput module (F), which can save results both locally and remotely.

We show a hypothetical workflow for this test case in Figure 1. The entire workflow has already been demonstrated (outside of Arbor) in a pilot study 29 using a virtual species on a spatially realistic matrix of 144 cells, and climate modeled in 130 time steps (back to 140,000 years before present). We ******will also be able to use the comparative capabilities of Arbor to constrain niche models to follow patterns expected if species’ response to abiotic factors follows evolutionary history (i.e., closely related species have niches that are more similar to one another than distantly related species). This will allow the user to directly test for phylogenetic structure in historic and extant species distribution patterns. We will use the New World genus *Aphelocoma* (scrub jays), which has 9 phylogenetic species, as a biological application of this test case. The phylogeny of the clade is well established 30 and predictions of Pleistocene ranges have been made 31, but without consideration to the historical trajectories of climatic factors and the phylogenetic history of the clade. We will use the new methods and algorithms implemented in Arbor to model scrub jay species distributions while incorporating paleoclimatic and phylogenetic data.

To include information about species’ niches in biogeographic models, we will use the *Lagrange* module to test hypotheses about the processes that govern range changes over time. Currently, the Lagrange program can fit process-based models of changes in species ranges through time by modeling speciation, extinction, and dispersal among a set of geographic regions. One can either assume that dispersal rates among these regions are constant or make modifications based on hypothesized patterns of geographic connections through time. The *Lagrange* module in Arbor will allow us to modify transition rates as a function of historical climate, directly testing whether geographic range shifts affect biogeographic processes. As a supplemental biological application, we will use this approach to investigate the diversification of *Heliconia* plants and their hummingbird pollinators. As these are Andean taxa that diversify during uplift activity, this test case will address the common biogeographical hypothesis that the Andean uplift and subsequent diversification of ecological niche space led to the *in situ* diversification of local lineages, and that current species distribution is largely driven by historical distributions and shifts in climatic factors 32.

## Arbor outreach

Our outreach and education efforts integrate four main goals to ensure that Arbor plays a meaningful role in advancing science and education in comparative evolutionary biology. As part of the development of Arbor, we will **(1)** create novel educational programs targeted to K-12 and the general public in collaboration with several public science centers; **(2)** explicitly involve ****diverse researchers and students, providing training both in software development and in using Arbor through hackathons and workshops; **(3)** engage undergraduates as well as graduate students and postdoctoral fellows in the development of Arbor; and **(4)** leverage and augment research conducted by other NSF-supported Tree of Life and Dimensions of Biodiversity projects.

We plan to develop a software interface, selected data sets, and lesson plans that will allow middle school, high school, and undergraduate students to engage with authentic scientific data as they develop tree-thinking skills. This interface will be a streamlined version of Arbor with a graphically appealing interface and more limited data analysis options to facilitate student use. Rich but appropriate data sets will be selected from the parent project so that students can explore a variety of evolutionary scenarios (e.g., co-diversification, ecological niches through time) and investigate multiple questions. Lesson plans will provide background information and explicit suggestions for instructors regarding how these materials can best be incorporated into classrooms to teach evolution, systematics, comparative biology, and the process of science.

## The Arbor team

The arbor team includes both biologists and computer scientists. Biologists include Bob Thacker (University of Alabama at Birmingham), Chelsea Specht (University of California, Berkeley), Jorge Soberon (University of Kansas), and Luke Harmon (University of Idaho). We span a range of biological expertise and interests, including symbiosis, genetics, developmental biology, species distributions, and comparative methods. Computer scientists include Charlie Hughes (University of Central Florida) and Curt Lisle (KnowledgeVis). We are also working closely with a team of programmers from KitWare, led by Wes Turner and Jeff Baumes. The computer scientists have extensive expertise with workflow software, visualization, user interaction and statistical analyses of large datasets.

## Community involvement: how you can get involved now with Arbor

Although the Arbor team is small now, we will grow more inclusive through a series of planned training courses and hackathons. Training courses will be geared towards teaching end-users how to obtain and analyze data using Arbor workflows, while hackathons will help programmers get up to speed and create new modules for Arbor. The first of these meetings will take place in late 2013. We will also be offering ‘sabbaticals’ to postdoctoral scholars who are actively generating phylogenetic and comparative data that could be analyzed within Arbor. These 3-6 month appointments allow postdocs to work closely with the Arbor team to integrate their data into Arbor and design novel Arbor workflows that are suited to their particular questions, thereby immediately developing the capacity of Arbor to serve the scientific community. We encourage community feedback and involvement at every step of the process; the goal of the Arbor team is to produce efficient and open-source software that will enable scientists, students, and educators to understand the Tree of Life in new ways. Everyone is free to provide comments or suggestions either here on the PLOS Currents page or via our website (http://www.arborworkflows.com/).

## Conclusion

We are rapidly approaching a “rough draft” for the Tree of Life. As phylogenetic trees become more reliable, larger, and more complete, the need for integrative approaches to analyze and understand those trees and their evolutionary significance becomes critical. Arbor will allow users to test key evolutionary hypotheses with methods that are new and flexible using massive amounts of data. Arbor’s most unique feature is allowing users to create sharable and citable workflows that combine methods in new and unanticipated ways. We are building the Tree of Life, but we do not yet know what it can tell us about the process of evolution. Arbor will allow us to harvest massive amounts of new information from the Tree of Life and facilitate new discoveries about organismal evolution.

## References

[1] Bininda-Emonds OR, Cardillo M, Jones KE, MacPhee RD, Beck RM, Grenyer R, Price SA, Vos RA, Gittleman JL, Purvis A. The delayed rise of present-day mammals. Nature. 2007 Mar 29;446(7135):507-12. PubMed PMID:17392779. 17392779 10.1038/nature05634

[2] Smith SA, Beaulieu JM, Donoghue MJ. Mega-phylogeny approach for comparative biology: an alternative to supertree and supermatrix approaches. BMC Evol Biol. 2009 Feb 11;9:37. PubMed PMID:19210768. 19210768 10.1186/1471-2148-9-37 PMC2645364

[3] Kattge J, Díaz S, Lavorel S, Prentice IC, Leadley P, Bönisch G, Garnier E, and 128 others. 2011. TRY - a global database of plant traits. Global Change Biology 17: 2905-2935.

[4] Jones KE, Bielby J, Cardillo M, Fritz SA, O'Dell J, Orme CDL, Safi K, Sechrest W, Boakes EH, Carbone C, Connolly C, Cutts MJ, Foster JK, Grenyer R, Habib M, Plaster CA, Price SA, Rigby EA, Rist J, Teacher A, Bininda-Emonds ORP, Gittleman JL, Mace GM, Purvis A. 2009. PanTHERIA: a species-level database of life history, ecology, and geography of extant and recently extinct mammals. Ecology 90: 2648.

[5] Guralnick R, Hill A. Biodiversity informatics: automated approaches for documenting global biodiversity patterns and processes. Bioinformatics. 2009 Feb 15;25(4):421-8. PubMed PMID:19129210. 19129210 10.1093/bioinformatics/btn659

[6] Stark C, Breitkreutz BJ, Chatr-Aryamontri A, Boucher L, Oughtred R, Livstone MS, Nixon J, Van Auken K, Wang X, Shi X, Reguly T, Rust JM, Winter A, Dolinski K, Tyers M. The BioGRID Interaction Database: 2011 update. Nucleic Acids Res. 2011 Jan;39(Database issue):D698-704. PubMed PMID:21071413. 21071413 10.1093/nar/gkq1116 PMC3013707

[7] Yatsunenko T, Rey FE, Manary MJ, Trehan I, Dominguez-Bello MG, Contreras M, Magris M, Hidalgo G, Baldassano RN, Anokhin AP, Heath AC, Warner B, Reeder J, Kuczynski J, Caporaso JG, Lozupone CA, Lauber C, Clemente JC, Knights D, Knight R, Gordon JI. Human gut microbiome viewed across age and geography. Nature. 2012 May 9;486(7402):222-7. PubMed PMID:22699611. 22699611 10.1038/nature11053 PMC3376388

[8] Moles AT, Ackerly DD, Webb CO, Tweddle JC, Dickie JB, Westoby M. A brief history of seed size. Science. 2005 Jan 28;307(5709):576-80. PubMed PMID:15681384. 15681384 10.1126/science.1104863

[9] Alfaro ME, Santini F, Brock C, Alamillo H, Dornburg A, Rabosky DL, Carnevale G, Harmon LJ. Nine exceptional radiations plus high turnover explain species diversity in jawed vertebrates. Proc Natl Acad Sci U S A. 2009 Aug 11;106(32):13410-4. PubMed PMID:19633192. 19633192 10.1073/pnas.0811087106 PMC2715324

[10] Meredith RW, Janečka JE, Gatesy J, Ryder OA, Fisher CA, Teeling EC, Goodbla A, Eizirik E, Simão TL, Stadler T, Rabosky DL, Honeycutt RL, Flynn JJ, Ingram CM, Steiner C, Williams TL, Robinson TJ, Burk-Herrick A, Westerman M, Ayoub NA, Springer MS, Murphy WJ. Impacts of the Cretaceous Terrestrial Revolution and KPg extinction on mammal diversification. Science. 2011 Oct 28;334(6055):521-4. PubMed PMID:21940861. 21940861 10.1126/science.1211028

[11] Rosindell J, Harmon LJ. OneZoom: A Fractal Explorer for the Tree of Life. PLoS Biol. 2012 Oct;10(10):e1001406. PubMed PMID:23091419. 23091419 10.1371/journal.pbio.1001406 PMC3472976

[12] Reichman OJ, Jones MB, Schildhauer MP. Challenges and opportunities of open data in ecology. Science. 2011 Feb 11;331(6018):703-5. PubMed PMID:21311007. 21311007 10.1126/science.1197962

[13] Ahrens J, Geveci B, Law C. 2005. ParaView: An End User Tool for Large Data Visualization. in The Visualization Handbook, C. D. Hansen and C. R. Johnson, Eds.. Elsevier: Burlington, MA.

[14] Goecks J, Nekrutenko A, Taylor J. Galaxy: a comprehensive approach for supporting accessible, reproducible, and transparent computational research in the life sciences. Genome Biol. 2010;11(8):R86. PubMed PMID:20738864. 20738864 10.1186/gb-2010-11-8-r86 PMC2945788

[15] Freckleton RP. Fast likelihood calculations for comparative analyses. Methods in Ecology and Evolution2012; 3: 940-947.

[16] Maddison WP, Maddison DR. Mesquite: a modular system for evolutionary analysis. 2011; Version 2.75 http://mesquiteproject.org.

[17] Paradis E, Claude J, Strimmer K. APE: Analyses of Phylogenetics and Evolution in R language. Bioinformatics. 2004 Jan 22;20(2):289-90. PubMed PMID:14734327. 14734327 10.1093/bioinformatics/btg412

[18] Harmon LJ, Weir JT, Brock CD, Glor RE, Challenger W. GEIGER: investigating evolutionary radiations. Bioinformatics. 2008 Jan 1;24(1):129-31. PubMed PMID:18006550. 18006550 10.1093/bioinformatics/btm538

[19] Kembel SW, Cowan PD, Helmus MR, Cornwell WK, Morlon H, Ackerly DD, Blomberg SP, Webb CO. Picante: R tools for integrating phylogenies and ecology. Bioinformatics. 2010 Jun 1;26(11):1463-4. PubMed PMID:20395285. 20395285 10.1093/bioinformatics/btq166

[20] Fitzjohn RJ. Diversitree: comparative phylogenetic analyses of diversification in R. In press, Methods in Ecology and Evolution.

[21] Schroeder, W., K. Martin, and B. Lorensen. 2006. Visualization Toolkit: An Object-Oriented Approach to 3D Graphics, 4th Edition. Kitware, Inc.: Clifton, NY.

[22] Schroeder W, Martin K, Lorensen B. Visualization Toolkit: An Object-Oriented Approach to 3D Graphics, 2006, 4th Edition. Kitware, Inc.: Clifton, NY.

[23] Wylie B, Shead T, Baumes J. Information visualization in VTK: the titan project. Tutorial presented at IEEE Visualization Conference, 2008; Available online at http://titan.sandia.gov/media/Information_Visualization_in_VTK.pdf

[24] Peterson AT, Soberón J, Pearson RG, Anderson RP, Martínez-Meyer E, Nakamura M, Araújo MB. Ecological Niches and Geographic Distributions. 2011; Princeton University Press, Princeton, NJ.

[25] Elith J, Leathwick JR. Species distribution models: ecological explanation and prediction across space and time. Annual Review of Ecology, Evolution, and Systematics 2009; 40:677-697. 10.1146/annurev.ecolsys.110308.120159

[26] Ree RH, Moore BR, Webb CO, Donoghue MJ. A likelihood framework for inferring the evolution of geographic range on phylogenetic trees. Evolution. 2005 Nov;59(11):2299-311. PubMed PMID:16396171. 16396171

[27] Ree RH, Smith SA. Maximum likelihood inference of geographic range evolution by dispersal, local extinction, and cladogenesis. Syst Biol. 2008 Feb;57(1):4-14. PubMed PMID:18253896. 18253896 10.1080/10635150701883881

[28] Ree RH, Sanmartin I. Prospects and challenges for parametric models in historical biogeographical inference. Journal of Biogeography 2009; 36:1211-1220.

[29] Barve NV, Barve V, Jiménez-Valverde A, Lira-Noriega A Maher SP, Peterson AT, Soberón J, Villalobos F. The crucial role of the accessible area in ecological niche modeling and species distribution modeling. Ecological Modelling 2011; 222: 1810-1819.

[30] Rice NH, Martínez-Meyer E, Peterson AT. Ecological niche differentiation in the Aphelocoma jays: a phylogenetic perspective. Biological Journal of the Linnean Society 2003; 80: 369-383.

[31] Peterson AT, Martínez-Meyer E, González-Salazar C. Reconstructing the Pleistocene geography of the Aphelocoma jays (Corvidae). Diversity and Distributions 2004; 10: 237-246.

[32] Rangel TF, Diniz-Filho JA, Colwell RK. Species richness and evolutionary niche dynamics: a spatial pattern-oriented simulation experiment. Am Nat. 2007 Oct;170(4):602-16. PubMed PMID:17891738. 17891738 10.1086/521315

[33] Lenards A, Merchant N, Stanzione D. Building an environment to facilitate discoveries for plant sciences. Proceedings of the 2011 ACM workshop on Gateway computing environments 2011; 51-58. 10.1145/2110486.2110494

